# Ethnozoological knowledge of traditional fishing villages about the anadromous sea lamprey (*Petromyzon marinus*) in the Minho river, Portugal

**DOI:** 10.1186/s13002-019-0345-9

**Published:** 2019-12-27

**Authors:** Heitor Oliveira Braga, Mário Jorge Pereira, Fernando Morgado, Amadeu M. V. M. Soares, Ulisses Miranda Azeiteiro

**Affiliations:** 1 Department of Biology & CESAM - Centre for Environmental and Marine Studies, University of Aveiro, 3810-19 Aveiro, Portugal; 2 CAPES Foundation, Ministry of Education of Brazil (BEX: 8926/13-1), Caixa Postal 250, Brasília, DF 70040-020 Brazil

**Keywords:** Ethnobiology, Ethnozoology, Local ecological knowledge, Diadromous fish, Cyclostomes

## Abstract

**Background:**

Sea lamprey (*Petromyzon marinus*) is a diadromous fish compromised by various stressors, which can lead to population decline and the urgency of stronger conservation regulation. In the absence of documentation of direct knowledge of local populations, a broader zoological and ecological understanding of sea lamprey fishing has become vital for the preservation of traditional practices and conservation of this migratory fish. To this purpose, we collected data from the *P. marinus* about the artisanal fisheries profile, folk taxonomy, habitat, reproduction, migration, and displacement using a low-cost methodology, through ethnobiology tools, in the four riverine fishing villages in Portugal.

**Methods:**

A total of 40 semi-structured interviews were carried out during the winter of 2019 in crucial fishing villages in the Minho river. Fishers were selected by random sampling and the snowball technique when appropriate. Interviews applied contained four parts (fisher’s profile, projective test, knowledge about fishing, and ethnozoological knowledge about the sea lamprey). Informal knowledge was analyzed following an emic-etic approach and the set-theoretical Union of all individual competences. The Code of Ethics of the International Society of Ethnobiology (ISE) was the main parameter for the conduction of this ethnozoological research and related activities in the Cooperminho project.

**Results:**

This first ethnobiological study of the sea lamprey (*Petromyzon marinus*) in Portugal showed a sample of predominantly male fishers, averaging 57.13 years old, and average fishing experience of 37.18 years. The average income of fishers is about 688.28 Euros, and the level of education was predominantly basic. Data from artisanal fisheries showed the time and frequency of fishing, the characterization of fishing boats, and general information on catching lamprey in the Minho river. Three new folk names were attributed to *P. marinus*. Fishers mentioned sites with rock fragments and sandy bottoms and depth ranges ranging from 0 to 8 m as likely sea lamprey habitats. The villages of Monção and Melgaço are the last areas of the river where you could spot sea lamprey, as well as the last probable spawning grounds for this fish in the Minho river. The hydroelectric dams and predatory fisheries were considered the main obstacles to the migration of sea lamprey. Finally, local fishers also shared the lamprey migration season to feed and spawn.

**Conclusions:**

Fishers shared a vast informal knowledge of sea lamprey zoology and ecology typical of anadromous species of the Petromyzontidae family, in the central traditional Portuguese communities on the Minho river. This fisher’s knowledge becomes essential to preserve cultural practices of the sea lamprey, which is currently highly susceptible to anthropogenic pressures. Given the real warning of population extinction in the Portuguese rivers (such as the Minho river) and a similar trend in Spanish territory, ethnozoological studies of sea lamprey in Spanish fishing communities may support our findings. Also, this study may assist in the adaptive participatory management of these anadromous fish, as well as in documentation of local ecological knowledge (LEK) and centuries-old fishing practices that are also vulnerable in modern times on the international frontier Minho river.

## Background

Lampreys have a deep ancestry shared with ours, being representatives of an ancient line of vertebrates that diverged about 500 million years ago [1]**.** The phylogenetic position of the lampreys, mainly due to the varied characteristics of the vertebrates present in the species, makes it an excellent model of experiments to understand the ancestry of vertebrate genomes better [1, 2]. Among all species of lamprey, the sea lamprey (*Petromyzon marinus* Linnaeus, 1758) stands out because it is the largest of all the lampreys of aquatic systems [3]. This cyclostome fish (Petromyzontidae) is distributed on both sides of the North Atlantic Ocean, occurring still in less abundance in some rivers of Poland, Southwest of England and Mediterranean Sea [4].

The sea lamprey is typically anadromous species in which it passes the initial stages of life in streams and the adult feeding stage in marine waters [5]. This species usually begins the migration to spawn between January and May, after the feeding phase, where parasites other marine mammals and teleost, in which they consume body fluids and tissues of these animals [3, 5, 6]. Populations of sea lampreys can be found in the main Portuguese watersheds, being more prominent in the central and northern regions [7]. There is a highlight of the abundance of *P. marinus* in the following hydrographic basins: Minho, Mondego, Lima, Tagus, Cávado, Guadiana, and Vouga (that includes the Lagoon of Aveiro) [8]. In the Portuguese rivers, the sea lamprey enters the estuary to begin their reproductive migration in late December and early January, with peaks in February and March, extending up to May and June, depending on weather conditions [4, 9].

In Europe, anadromous populations of sea lamprey tend to be threatened by several stressors, which may lead to population decline, and a need for more robust conservation regulation [2]. This species have been listed as “Vulnerable” on the OSPAR List of threatened or declining species and habitat [10], and in the Red Book of Vertebrates of Portugal [11]. Particularly in the native range, *P. marinus* also was considered threatened in some countries such as Spain, France, and Portugal [12]. Notwithstanding, in the last classification of the IUCN Red List Category and Criteria, the sea lamprey presents as Least Concern, as a stable population [13].

Riverside populations in Portugal have used the sea lamprey as a fishing resource for several centuries, mainly through the capture with drifting trammel nets, large fyke nets and the use of “*Pesqueiras*” (traditional local traps) [8, 14]. Additionally, this diadromous fish is still highly recognized for its considerable commercial and economic value, supporting the local fishing in these Portuguese riverine systems, highlighting here the Minho river in the North of Portugal [15]. However, in the last decades, there has been a population decline of this natural resource, mainly due to overfishing, construction of insurmountable hydroelectric dams, extraction of aggregates, and loss of water quality that directly generate impacts on spawning habitats and larvae [4, 14].

Due to all these peculiarities of the local communities [8, 14, 15] and conservation of sea lamprey in Portugal [10–12], a study that seeks to understand how these local communities identify this biological resource and classify it can collaborate with information on the conservation and zoology of this cyclostome in the Minho river. In this context, we have ethnozoological studies whose main objective is to understand how the different people of the world perceive and interact with the resources of the fauna, with combinations of elements from the natural and social sciences [16]. This hybrid discipline also thoroughly investigates the environmental, anthropological, sociological, economic, and historical factors of the relationship between humans and animals [17]. Finally, Marques [18] defines ethnozoology as the transdisciplinary study of thoughts and perceptions (knowledge and beliefs), feelings (affective representations), and behaviors (attitudes) that mediate the relations between the human populations that possess them with the animal species of the ecosystems that include them.

Studies with ethnozoological characters are still scarce in Portugal. The only published works in the same field of ethnoscience on the local ecological knowledge (LEK) are from the European sardine in Peniche, Portugal [19, 20], and another contribution about fisher’s knowledge about the understanding and identification of Essential Fish Habitats (EFH) for skate species in central of Portugal [21]. Elsewhere in Europe, there are only a few social and cultural studies targeting anadromous fish [22–27], but nothing similar to this research, and with LEK attributes around the sea lamprey (*P. marinus*). Therefore, this paper was a pioneering study on the ethnozoology of an important anadromous migrant fish (*Petromyzon marinus*) in the Minho river, Portugal. We sought to share data from the fisher’s profile, artisanal fisheries, folk taxonomy, habitat, reproduction, migration, and displacement provided by local riverine people from the sea lamprey of this frontier river in the Iberian Peninsula.

## Methods

### Study site

**Fig. 1:**
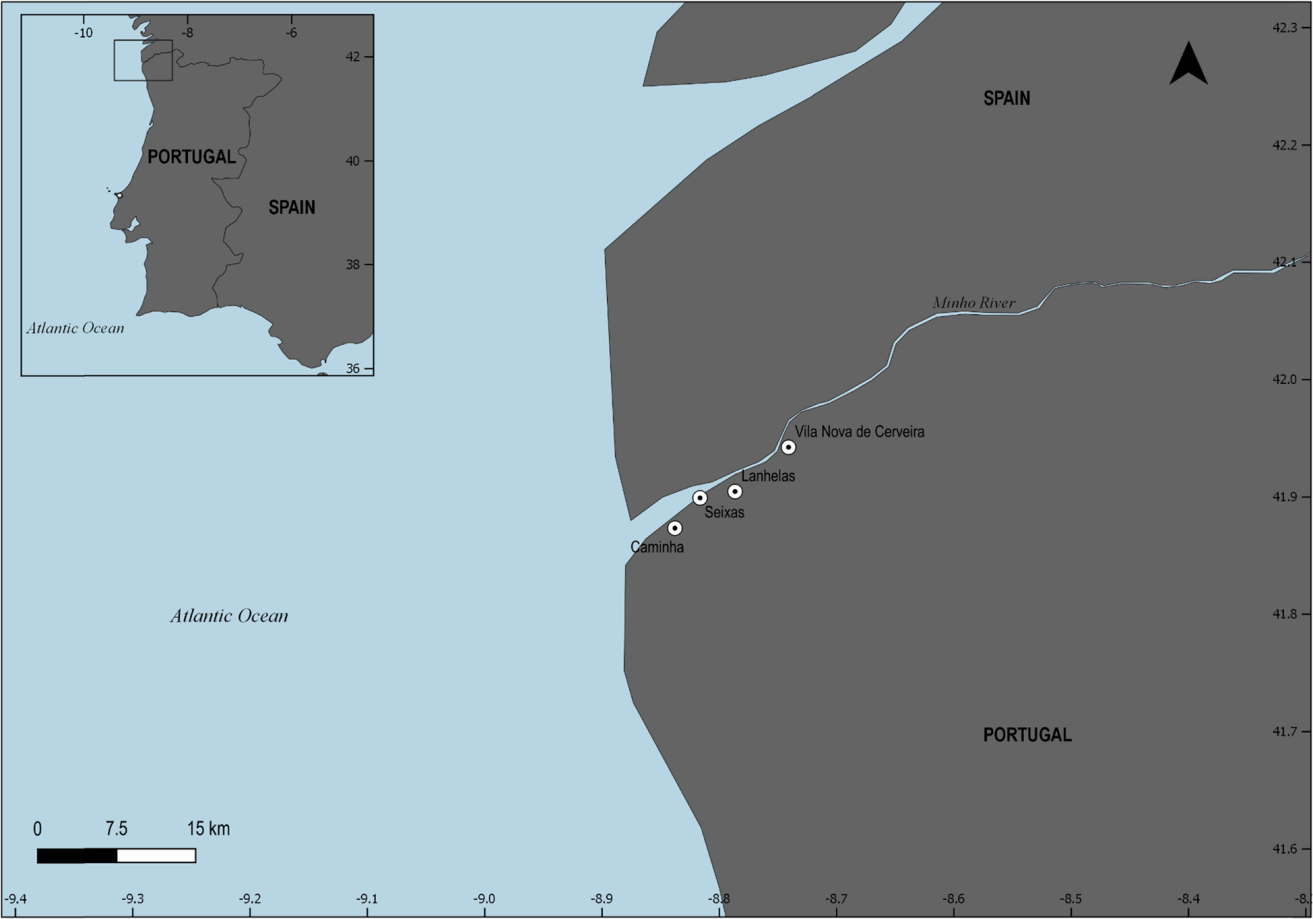
Map showing the four main Portuguese fishing villages of the Minho river, Iberian Peninsula (Caminha, Seixas, Lanhelas, and Vila Nova de Cerveira). Credits: J. Musiello-Fernandes

This study was carried out in the leading traditional Portuguese fishing communities of the river Minho (Fig. 1). This International river, also called *Miño* in the Spanish territory, presents about 300 km from its source in Spain (50 km north of Lugo in Galicia, Spain), and in the last 75 km, this aquatic system establishes a border region between Portugal and Spain [28]. This hydrographic basin presents about 17.080 km^2^ (5% located in Portugal) and drains in the North Atlantic with an annual average discharge of 300 m^3^ s^−1^ of freshwater [29]. In this area of study, the climate is a temperate sub-Mediterranean ocean and presents temperatures around 12–17.5 °C and average annual rainfall between 700 and 1300 mm [30]. This frontier river also includes the list of the main sites of Community importance (SIC: PTCON0019) located in Portuguese national territory and belonging to the Atlantic biogeographical region, constituting an area of the great ecological significance of the European Union by the Natura 2000 Network [31]. Additionally, the Minho river is also known for its enormous cultural value in the Iberia Peninsula [32].

The fishing villages sampled along the Minho river were Caminha (41° 52′ 38.49″ N, 8° 50′ 19.82″ W), Seixas (41° 53′ 56.26″ N, 8° 48′ 59.21″ W) Lanhelas (41° 54′ 28.04″ N, 8° 47′ 26.72″ W), and Vila Nova de Cerveira (41° 56′ 34.24″ N, 8° 44′ 26.89″ W). These Portuguese riverside communities are recognized as the most crucial fishing villages of the Minho river [33]. Caminha is the municipality that is located near the mouth of the estuary of the River Minho and presents an estimated population of 16,684 inhabitants and territorial area of 136.52 km^2^. Seixas has 1502 inhabitants and has an area of about 883 km^2^. Lanhelas has about 991 inhabitants and an area of 504 km^2^, and Vila Nova de Cerveira has 9553 inhabitants and an area of 108.47 km^2^ [34]. In this region, the following fishing associations are distinguished: Association of Fisheries Professionals of the Minho river and the Sea and the Ribeira Minho Fishermen’s Association. The Port Authority of Caminha, which is one of the observatories of the Portuguese Maritime Heritage Register, indicates 163 fishing boats enrolled in the Minho river in 2019. Ultimately, the number of fishers reach 350 from the mouth of Caminha to the city of Valença in Portugal.

### Interviews and data analyses

Semi-structured interviews [35] were carried out during the winter of 2019, within the allowed fishing period of the sea lamprey (*Petromyzon marinus*, Linnaeus 1758, Fig. 2) in the River Minho. The researcher responsible for collecting data has established himself in one of the fishing villages (Lanhelas, Portugal) to create an environment of trust with the local population, which is quite peculiar and closed. Initially, successive visits also were made to the fisher’s meeting places and fishing ports so that the researcher could create a trustworthy atmosphere in all fishing villages.

**Fig. 2:**
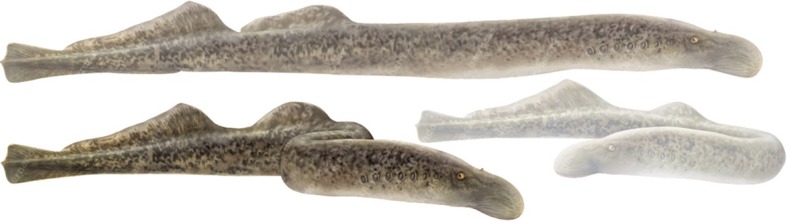
Image of the ethnozoological object of study (sea lamprey) carried out in the riverside villages of the Minho river. Credits: F. Correia (aut.: C. Barrocas)—Scientific Illustration Laboratory of the University of Aveiro, Portugal

After this first step, the interviewees were randomly selected at the main meeting places of the sea lamprey fishers in the four traditional communities. When possible and convenient, the “snowball technique” [36] was used to increase the sample of fishers in the region. In this method, the respondent or group of key informants led to other individuals who possess relevant information about the sea lamprey.

Before each interview, the interviewee received a Statement of Informed Consent (IC), in which there was information about the purpose of the research, the responsible institution, the confidentiality agreement of the data provided and the address of the social media (Facebook and Instagram—@cooperminho) for possible doubts, clarifications, and follow-up of the Cooperminho project research. In Portugal, there is no ethical committee approval needed. However, this present study followed the standards of ethics of the International Society of Ethnobiology (ISE) [37].

Standardized interview script applied in the fishing villages was composed of four parts (Additional file 1). The first part of the interview contained information on the variables of the fishers interviewed (gender, name, age, schooling, and locality). In the second part, there was a projective test [38] where a colored image of the sea lamprey in a mobile electronic device was presented to the fishers for identification of the species. In the third part, there were questions about knowledge about fishing (time of experience, fishing association, income, and fishing frequency). And in the last part of the interview script, the researcher sought to collect data on fisher’s ethnozoological about the sea lamprey (Fig. 1) in the Minho river, Portugal. In this step, it contained questions about the folk taxonomy, habitat, reproduction, and migration of this anadromous fish.

Qualitative and quantitative data from interview transcripts provided by fishers were organized and entered Microsoft Excel Office 365 MSO for further analysis using descriptive statistics techniques as a form of quantitative approach. Traditional ichthyological knowledge was analyzed through the construction of comparative cognitive tables in which the local native information is compared with the published literature [39, 40]. Informal knowledge data also followed an emic-etic approach [41], and the set-theoretical Union of all individual competences, in which the notion of the “omniscient informant” is employed [42].

## Results

### Fisher’s profile

Interviews were conducted with fishers at Caminha (*N* = 13), Seixas (*N* = 6), Lanhelas (*N* = 14), and Vila Nova de Cerveira (*N* = 7) to report the local knowledge on sea lamprey in the Minho river (Table 1). Only one interviewed was a fisherwoman. The age of the interviewees ranged from 32 to 88 years, with an average of 57.13 (± 14.76) years. The level of education was classified into Portuguese four classes: Primary education, A = first cycle (1–4 years), *N* = 14; B = second cycle (5–6 years), *N* = 8; C = third cycle (7–9 years), *N* = 10; and Secondary education (10–12 years), *N* = 5; and the Higher education (more than 12 years), *N* = 3.

**Table 1:** Summary of the demographics of the Minho river fishers (*N* = 40)

Variables	*N* of fishers		
*Fishing village interviews*			
Caminha	13		
Seixas	06		
Lanhelas	14		
Vila Nova de Cerveira	07		
*Gender*			
Female	01		
Male	39		
*Education level (years of schooling)*			
1st cycle (1–4 years)	14		
2nd cycle (5–6 years)	08		
3rd cycle (7–9 years)	10		
Secondary education (10-12 years)	05		
Higher education (more than 12 years)	03		
*Members of the fishing association*			
Fisheries professionals of the Minho river and the sea	12		
Ribeira Minho	06		
Others	03		
Not associated	19		
*Other complement professions*			
(civil construction, tourism, and local commerce)	28		
*Fisher’s activity*			
Active	30		
Retired	10		
	Minimum and Maximum	Average	Standard Deviation (sd)
*Age (years)*	32 - 88	57.13	± 14.76
*Income (Euros)*	300 - 1500	688.29	± 271.35
*Fishing experience (years)*	02 - 70	37.18	± 17.42

The average fishing experience was 37.18 (± 17.42) years. Of the total of fishers interviewed (*N* = 40), 30 are active, and ten are retired. These retired interviewees have had a long fishing experience in the past and still remain in direct contact with active fishers at informal gatherings in the fishing villages. Fisher’s income varied from 300 Euros to 1500 Euros, with an average of 688.29 (± 271.35) Euros. Twenty-eight (*N* = 28) fishers complement their income with other economic activities such as civil construction, tourism, and local commerce. Part of the respondents (*N* = 21) are enrolled in a local fisher’s association (Minho river and Association of Sea Fishing Professionals, *N* = 12; Ribeira Minho Association, *N* = 6; and other associations, *N* = 3).

### Lamprey artisanal fisheries

Fisher’s knowledge showed that lamprey catches occur between January and April and reported that fishers usually fish this anadromous species up to seven times a week (*N* = 20) during this season in the Minho river. Another seventeen fishers (*N* = 17) fish lamprey five to six times a week. Finally, two interviewees (*N* = 2) said to fish three to four times a week, and only one said to fish once a week. Fishing time per day varies from 1.5 to 9.5 h, with an average of 4.28 (± 1.54) hours depending on the weather conditions.

**Fig. 3:**
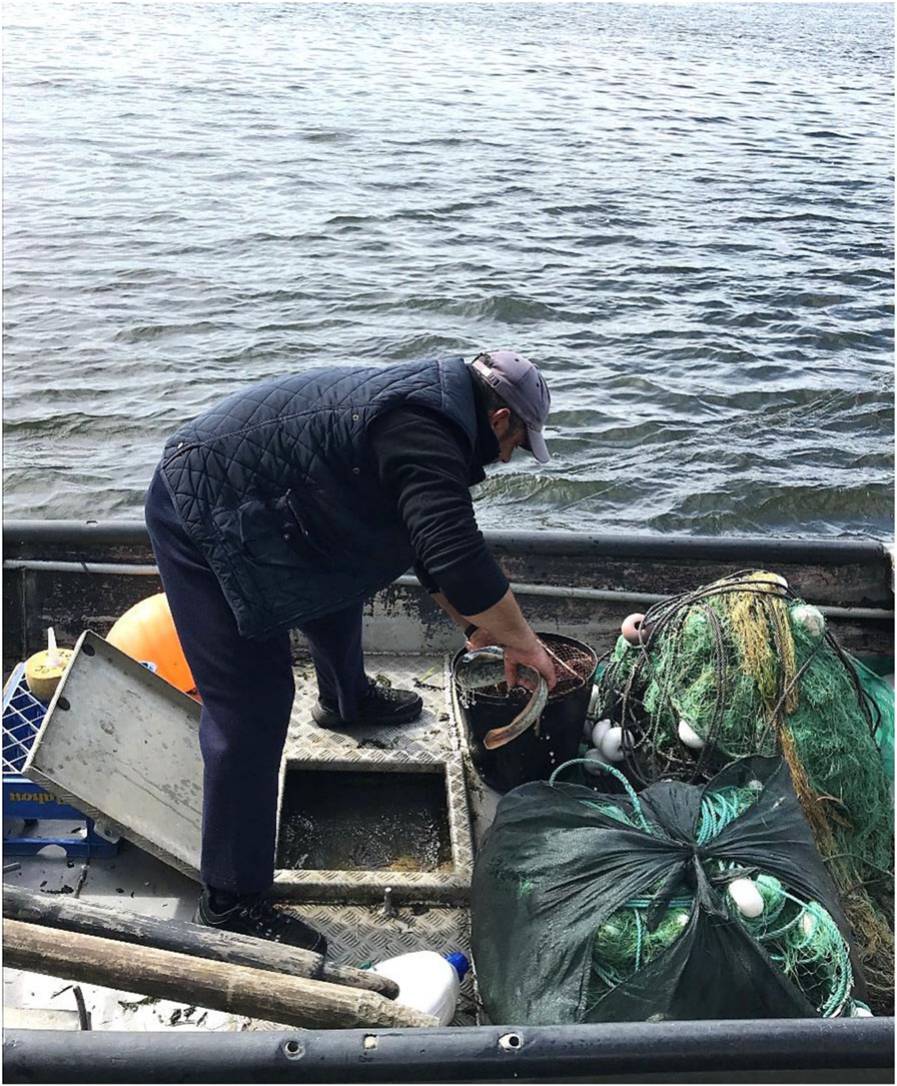
Typical sea lamprey fisher after a fishing trip operated by a small inland boat (< 6 m), through drifting trammel nets (*lampreeira*) in the fishing village of Lanhelas, Portugal. Photo: Braga. H.O. (Images published with the prior consent of participants)

This fishing schedule also varies according to the tides, being predominantly two times a day (day and night) (*N* = 40). Four other fishers (*N* = 4) added that fishing hours also vary according to the light of the moon. Fishing boats currently used according to the fishers interviewed are the fiber boat (16 citations), the aluminum boat (12 citations), and stainless-steel boat (1 citation). Rowboats were also mentioned by the other fishers (wooden boats in general: 12 citations). Among them, we have *Carocho* in Lanhelas Village (*N* = 4) and *Gamela* in Caminha Village (*N* = 4). In the fishing of the sea lamprey, they usually ship from one to two crew members per boat (one per boat: *N* = 15; two per boat: *N* = 19; one to two per boat: *N* = 6) and the size of these lamprey fishing boats varies between 4.0–5.6 and 0.95–3 m. Finally, the fishing gear used by Portuguese fishing villages for this fishing is locally called *lampreeira* (*N* = 40, Fig. 3).

### Folk taxonomy

All the fishers (*N* = 40) of the Minho river had success in the projective test. Thirty-nine (*N* = 39) named the adult species as lamprey. One of the interviewees also called the adult species of the same projective test as *Mouca* or lamprey. The names attributed to *P. marinus* in the juvenile stage were Little lamprey—*Lampreia pequenina* (*N* = 22), lamprey (same as the adult stage, *N* = 18), Crazy lamprey—*Lampreia louca* (*N* = 3), and *Sapeira* (*N* = 1). Some local proverbs are worth highlighting in this part as:

1. “The crazy are little lampreys. These do not develop. They are called "crazy" because they live buried and cannot move. A percentage of them stay in the river. These lampreys have no guidance power to go to sea. I use “crazy” lampreys as bait for sea fishing.”
2. “Small lamprey swims in the fishing season of this animal in Minho river. They are 10 to 15 cm long.”
3. “The small lamprey is buried in the river. There are many 5 cm long lampreys in Minho river.”

### Preferred habitat

The mean sea lamprey habitats (Table 2) are rock sites (18 citations), pebble stones—“*Seixos*” (10 citations), sandy bottoms (9 citations), freshwater sites (4 citations), and the Sargasso Sea (3 citations). Fishers also mentioned surface sites, depth sites, calm waters and crystal-clear waters (two citations each), and green backgrounds (laurel) (one citation). Artisanal fishers of Minho river also cited four preferred depth ranges of the sea lamprey, which ranged from 0 to 8 m. The depths fields are 0–2 m (*N* = 6), 0–4 m (*N* = 25), 0–6 m (*N* = 3), and 0–8 m (*N* = 1).

**Table 2:** Local Ecological Knowledge (LEK) about the sea lamprey habitat (*N* = 40)

Mean sea lamprey habitats	Citations	Depth ranges (m)	Fishers (*N*)
Rock sites	18	1. 0–2 m	06
Pebble stones—“Seixos”	10	2. 0–4 m	25
Sandy bottoms	09	3. 0–6 m	03
Freshwater sites	04	4. 0–8 m	01
Sargasso sea	03	Do not know	05
Surface sites	02		
Depth sites	02		
Calm and crystal-clear waters	02		
Green backgrounds (laurel)	01		
Highest number of juveniles in the Minho river (villages)	Citations	Juvenile sea lamprey (sites)	Citations
Monção	19	Northernmost areas of the river	15
Melgaço	12	Whole river	10
Valença	05	Banks of the river	02
Vila Nova de Cerveira	03	Streams of the river	02
Caminha (Coura river)	03	Gullies of the river	02
Gondarém	02	Muddy regions	01
Lapela (Monção municipality)	01		
A Torre, Ourense (Frieira Dam)	01		
Adult sea lamprey (sites)	Citations		
Whole river	15		
Minho river Mouth/“Barra de Caminha”	08		
Monção (village)	03		
Seixas to Vila Nova de Cerveira (villages)	03		
Melgaço (village)	01		

Fishers cited Monção (19 citations) and Melgaço (12 citations) as the regions with the highest number of juveniles in the Minho river. Interviewees mentioned the Valença region (five citations), Vila Nova de Cerveira (three citations), and Caminha (Coura river specifically; three citations). Finally, Fishers cited Gondarém (two citations), Lapela (Monção Municipality), and Frieira hydroelectric dam (Galicia, Spain) (one citation each). Some members of the fishing community also said that there were “little sea lampreys” in the whole river (10 citations), and the banks, streams, and gullies of river Minho (2 citations each). Fishers also mentioned the northernmost areas of the Minho river (15 citations) and the muddy regions of this river (1 citation). Finally, the adult fish inhabit the whole river (32 citations), especially the Minho river Mouth/“Barra de Caminha” (8 citations). Other said they had found more adult lampreys in Monção (three citations), and from Seixas to Vila Nova de Cerveira (three citations) and even Melgaço (one citation) (Table 2). We can highlight some passages recorded during interviews in the traditional communities of the Minho river about the preferred habitat of the sea lamprey:

1. “The lamprey rests in dark places and some specific locations. When the tide changes, it moves (The center of the moon is what makes them change).”
2. “If there is hot water, the adult lamprey enters the river more.”
3. “Everywhere there are juvenile lampreys. But there are more young individuals upstream. Here in the region of Lanhelas and Gondarém, there are also some of these! But there are more in the Monção area and near the hydroelectric dam.”
4. “There are juvenile lampreys throughout the Minho river. The little lamprey abounds in December when local fishers catch the small eel. These lampreys pass through all the parishes around the river Minho, especially in the region of Monção.”

### Migration

Migratory movements (Table 3) occur mainly from the mouth of the Minho river towards the upstream (*N* = 23) so that the sea lampreys seek to spawn and complete the life cycle. However, other fishers (*N* = 16) said that this species moves both upstream and downstream. No fisher has pointed out that fish move only from upstream to downstream. Only one interviewee did not know the information about this topic. Fishers also detailed the likely areas of the Minho river that sea lampreys can reach during their migration. The maximum points that the sea lamprey can move after entering the mouth of the Minho river are the Portuguese villages of Melgaço (*N* = 21) and Monção (*N* = 10). Specifically, other fishers mention the localities of São Gregório (Melgaço Municipality, *N* = 4), Lapela (Monção Municipality, *N* = 2) in Portugal, and the city of Ourense (*N* = 2), and Illa Fillaboa (next to river Tea, *N* = 1) in Galicia, Spain. Interviewees (*N* = 16) in the riverside communities of the Minho river also cited the hydroelectric dams as the last point of appearance of the lamprey in this river. Among these respondents, four fishers (*N* = 4) particularly cited the Frieira hydroelectric dam, which is in a border region (A Frieira, Pontevedra, Spain, and Cevide, Cristoval Parish in Melgaço, Portugal).

**Table 3:** Local Ecological Knowledge (LEK) about the migration of the sea lamprey (*N* = 40)

Direction of migrations	Fishers (*N*)	Maximum points of movement towards the upstream (villages)	Fishers (*N*)
Towards the upstream	23	Melgaço	21
Moves both upstream and downstream	16	Monção	10
Do not know	01	São Gregório (Melgaço Municipality)	04
		Lapela (Monção Municipality)	02
		Ourense	02
		Illa Fillaboa	01
Ways to get around barriers to migration	Citations	Migration delay factors	Citations
Swimming	32	The strength of the water current	11
Resting and leaning on the river rocks	07	The resting of the animal on rocks and other refuges	10
Breaking barriers instinctively	04	Hydroelectric dams	07
Parasitizing other fish	04	Predatory fishing	07
		Freshwater scarcity	04
		River flooding	04
		Do not know	01
Preferred time to migrate	Citations	Food migration (months)	Citations
Any time of the day	32	May	08
Nocturnal period (Preference)	10	November	07
According to tidal variation	07	December	04
Just the Night	04	June	03
According to the phases of the moon	03	January	02
Morning	01	April, July, August, and September (each one)	01
According to the arrival of the lamprey at the mouth of the river	01		
Do not know	01		

The delay or impediment in the movement of this anadromous fish along the Minho river may be due to some factors: the strength of the water current (11 citations), the resting of the animal on rocks and other refuges (10 citations), the hydroelectric dams of the river and predatory fishing (7 citations each), and freshwater scarcity and river flooding (4 citations each). Only four fishermen did not answer this question. In this same part of the interview conducted in the local communities of Minho, there is the report that sea lampreys circumvent this situation mainly breaking barriers and currents through swimming (32 citations). Fishers also pointed out that this animal overcomes obstacles by resting and leaning on the river rocks (seven citations), instinctively deviating natural and artificial barriers during displacement (four citations) and migrating with the aid (parasitism) of other fish (four citations), such as the Allis shad—*Alosa alosa* (Linnaeus, 1758).

Riverine fishers also shared the preferred time for the sea lamprey to migrate to the Minho river. According to fishers (32 citations), the lamprey migrates at any time of the day, but this animal prefers the nocturnal period (10 citations). Fishermen (seven citations) said that this migration occurs according to tidal variation, and four others (four citations) said it only happens at night. Respondents (three citations) also noted that movement occurs according to the phases of the moon, and only one fisherman said that the preference of the fish was for the morning (one citation). Finally, one fisher said that the lamprey’s preferred time to migrate was according to the arrival of the lamprey at the mouth of the river (1 citation), and another respondent could not answer this question (1 citation). Fishers mentioned the months of May (eight quotes), November (seven quotes), December (four quotes), June (three quotes), and January (two quotes) as the months that the lamprey migrates to feed. Interviewees also remembered the months of April, July, August, and September (one quote each). In this section about *P. marinus* migration, we can highlight some citations of the interviews as:

1. “Lampreys move back and forth and are more likely to migrate toward the source of the river.”
2. “Fish migrate upstream to spawn. They look for freshwater. It is what makes the fish spawn. More freshwater, the more they drop their eggs and die, just like the salmon.”
3. “The hydroelectric dams do not let the lamprey rise higher. They migrated to Ourense, Spain, when there were no dams.”
4. “Lampreys migrate both night and day. Mostly at low tide.”
5. “The fish goes downstream to feed in October and November. In the activities of glass eel (meixão) fishing, there are many lampreys. This fish has resistance.”
6. “Lamprey swims in deep water. They are good swimmers. This fish crawl through the sand like a snake.”
7. “Lamprey migrate clinging to other fish such as Allis Shad and other large fish.”
8. “The lamprey is very strong and easily transposes the forces of the currents.”

### Reproduction

All fishers (*N* = 40) reported that the sea lamprey has different sexes (males and females). However, they could not visually differentiate the sexes from individuals of the same species. Regarding reproductive behavior in this species, most fishers did not know which genus of lamprey sought the opposite sex to mate (*N* = 21). Nine interviewed (*N* = 9) said females found males to reproduce, while nine other fishers (*N* = 9) pointed out that males found females to fertilize their eggs. Only one respondent said there were no rules for this reproductive behavior (Table 4). Data from ethnozoological interviews conducted in the crucial fishing villages of the Minho river provided the likely zones and districts where the lamprey spawns in this aquatic system (Table 4). We can highlight here the villages of Melgaço (26 quotes) and Monção (18 quotes) located in the Viana do Castelo District along the Minho river. Some fishers also reported spawning of this anadromous fish in the village of Valença (nine quotes) and Vila Nova de Cerveira (five quotes) also belonging to the Viana do Castelo District. Areas of the Minho river above and near the hydroelectric dams, and in general areas of the upstream (reference point: interviewed fishers from Caminha to Lanhelas), are also likely spawning sites of the sea lamprey in this river.

**Table 4:** Highlights of local ecological knowledge (LEK) on sea lamprey reproduction (*N* = 40)

Reproductive behavior	Fishers (*N*)	Spawning areas (villages)	Citations
Females found males to reproduce	09	Melgaço	26
Males found females to reproduce	09	Monção	18
No rules for this reproductive behavior	01	Valença	09
Do not know	21	Vila Nova de Cerveira	05
Breeding season (months)	Citations	Lamprey death season (Months)	Citations
April	28	April	15
May	23	May	18
January	10	June	11
June	09		
March	07		
February	06		

The time of year that lampreys build their nest and reproduce according to interviewees varies from December to August (Table 4). April (28 citations) and May (23 citations) were the most remembered by fishers about the reproduction of this anadromous fish in the river Minho. Other interviewees also included the months of January (10 citations), June (9 citations), March (7 citations), and February (6 citations). When considering the month intervals mentioned by fishers, we can highlight April to May (*N* = 6), January to April (*N* = 5), April to July (*N* = 4), and end of April to May (*N* = 4).

All interviewees (*N* = 40) stated that the sea lamprey migrates from the sea to the Minho river when they are ready for reproduction. When asked if the adult lamprey returned to the same places where they were young or where they were roe, 32 fishers said yes, five said no, and three said they were not sure. Fishers highlighted April (15 quotes), May (18 quotes), and June (11 quotes) as the significant months when the sea lamprey begins to die on the Minho river (Table 4). More than half of interviewed (22 quotes) still said the death of this diadromous fish occurs after spawning. The main statements of the interviews about the reproduction of *P. marinus* were:

1. “Warmer waters are better for breeding. There is not much fish near the hydroelectric dams.”
2. “Poachers catch lampreys in Monção. They still wear a wetsuit. These fishers subsequently used the technique of smoking and effectively drying these fishery products.”
3. “When the river water gets hot, the lamprey spawns.”
4. “In May, the sea lamprey spawning is quite remarkable. This fish comes in afflicted to spawn. This animal has little care and is very exposed at that time and appears on the surface of the waters.”
5. “They reproduce higher on the banks of rivers. Lampreys make holes, dig the banks of streams into the ground and lay their eggs.”
6. “There is a tendency for lampreys to return to the same place where they were spawned, to spawn as well. One of them may fail.”
7. “They probably return as adults after 6 to 7 years at sea to die in the river. They spawn where their parents died.”
8. “After the lamprey spawning, it dies. The fish starts to get thin, bloodless, and dry.”
9. “Both female and male fish die a few days after spawning. They only do one cycle.”

## Discussion

### Fisher’s profile and lamprey artisanal fisheries

In Caminha village, there are about 50 boats engaged in fishing for the sea lamprey and eel juveniles—*Anguilla anguilla* (Linnaeus, 1758) [43]. According to the president of the local fishers’ association, in summer, this number drops to only 20 fishing boats that live exclusively other fish species [43]. In the Minho river, about 160 boats in 2018 were able to fish for sea lamprey [44], which is usually practiced by one crew member per fishing boat. Thus, we concluded that the sampling performed (*N* = 40) was more than enough to represent all the ethnozoological knowledge of the four main fishing communities of the Minho river. Among these interviewees, we highlight here the first documented interview of ethnozoological character with a woman (C.A, 46 years old, from Lanhelas village) in Portugal.

The average age of fishers was approximately 57 years old, following the same trend observed in an ethnobiological survey conducted in the Peniche fishing community in central Portugal (average of about 58 years old) [19, 20]. Minho riverine fishers showed a global trend observed in the profile of worldwide artisanal fishers where the level of education these individuals tends to be predominantly basic [45–47]. The fishing experience was about 38 years, which means that the interviewees have a long period of observation of the environment that surrounds them, as well as significant experience with the variations of sea lamprey fishing. Chan et al. (2019) point out that a fisher with a substantial fishing experience like this may be an essential factor in better understanding the trend of fish abundance in aquatic systems [48].

Sea lamprey fishing on the international section of the Minho river occurs mainly between January and June (mostly between February and April) [8]. In our findings, fishers drew attention to January, February, and April. After this season, some fishers often seek for other professional activities as noted in 28 interviews, in which we highlight construction, local commerce, and tourism. Also, there is a demand for other alternative professions, such as crew in sea fishing and agriculture [8]. The national minimum wage in Portugal is 600 Euros, which is lower than the 688.29 (± 271.35) Euros of the fishers interviewed, which should probably be complemented by the activities as mentioned above.

As for lamprey fishing in the Minho river, we highlight the *Pesqueiras* and the gillnet called *lampreeira.* The *Pesqueiras* is defined as “fixed fishing constructions on the stretch of river between the line that passes through the towers of the Lapela Castle (Portugal) and the church of Porto (Spain) and the upper boundary of the borderline.” [49]. These fixed constructions are distributed along the riverbanks, in different altitudes or even in the middle of the river [50]. On the other hand, the *lampreeira* is another type of drifting trammel net used predominantly in Iberian rivers [50]. In our findings, we also found this pattern of lamprey fishing gear used in the sampled communities (*N* = 40).

Even with the renewal of the fishing fleet in these Minho villages over the decades, there were still two active fishers (*N* = 2) from Caminha Village who said they used the *Gamela* boat. Although cited by some interviewed (*N* = 4), the traditional *Carocho* wooden boat was only portrayed as being used in the past, especially in the 1960s. This fact is in line with what was stated by Mendes (2008), who stresses the end of the use of the *Carocho* boat in Caminha at the same time, and the resistance of the use of the *Gamela* boat in this fishing community [51]. The purpose of this boat can still be useful today because it is more flexible and lighter, with ambivalent characteristics for both the river and the sea, near the mouth of Minho [51]. Lastly, there is a disconnection of the use of traditional boats from the river Minho in favor of a more modern and resistant fleet mainly for increasing the foraging area of this fishing limited to a specific season.

### Folk taxonomy

Folk taxonomy seeks to understand how people identify, classify, and name living organisms to understand better the basic principles of categorization and nomenclature, as well as the taxonomic, semantic, and linguistic criteria used in these procedures [52]. In our study, the projective test was used to support and confirm that the following questionnaire responses were being performed for the same species and to verify that fishers correctly identified the target species name, giving more reliability to all subsequent procedures. In this part of the interview, all fishers (*N* = 40) were successful, so no samples needed to be discarded in the data analysis phase.

Fishers during the interviews named *P. marinus* (adult stage) as the simple primary lexeme: lamprey. Others interviewed (*N* = 3) called this fish species by the composite primary lexeme: crazy lamprey—*Lampreia louca*, where “crazy” refers to the disordered and undirected behavior of this animal in some instances. Fishers also named the sea lamprey by the simple primary lexeme: *Sapeira*. In the juvenile phase, there was the name of this anadromous fish by *Mouca* (simple primary lexeme). In these last two cases, there was no explanation for the reason for this name for this lamprey species. It is also noteworthy that the Little lamprey—*Lampreia pequenina*, composite primary lexeme was predominant among juvenile lamprey. The term *pequenina* was attributed to this fish species because they are shorter in this stage of the life cycle. This folk taxonomy reported by riverine fisheries of Minho was classified according to Mourão [53]. Finally, the folk names *Lampreia louca*, *Mouca*, and *Sapeira* for *P. marinus* shared in this study are new to the scientific literature, which were previously designated in Portugal only by *Lampreia* (lamprey), *Lampreia do mar*, *Lampreia marinha*, and *Lampreira marinha* (sea lamprey) [54].

### Ethnozoology of sea lamprey

Lamprey larvae or ammocoete can commonly occur in running water as well as sometimes in gravel and mud banks [55]. The nests of this diadromous fish are usually found in shallow water between the gravel and the sandy bottom, often located above rapids or below buildings such as hydroelectric dams and bridges in calmer waters [55]. Pacheco (2013) also cites the rocky bottom as one of the preferred sites for the sea lamprey to get around the Minho river [56], where these fish can usually rest during the migration period [57]. Fishers from the same perspective also mentioned these rock fragments that encompass rounded stones called pebbles (*Seixos*) and sandy bottoms as a probable habitat of this species in this aquatic system. Additionally, with less citation but according to the scientific literature [4, 55–57], sites with calmer, crystalline and shallower waters, and bank muds were also cited as the preferred environments of one of the stages of the sea lamprey life cycle.

The depth stratum used by the adult sea lamprey species does not show significant seasonal differences over the seasons in the Great Lakes of North America [58]. In summer, this depth layer may vary more from 0 to 2 m, while during others season of the year the depth may be more concentrated within ranges of 2 to 5 m, 5 to 10 m, or more than 10 m in this river [58]. As for the larvae of these diadromous fish, they usually remain in the sludge at depths below 2 m or more in the one western European basin [59]. In the ocean, this species of fish is likely to forage in deep waters that can reach up to 4099 m [10]. Respondents similarly in parts report that the sea lamprey of the Minho river prefers to move mainly between 0 and 4 m and considers it a typical shallow-water fish.

The main fishing villages of the Viana do Castelo district from downstream to the river upstream are Caminha, Vila Nova de Cerveira, Valença, Monção, and Melgaço. Of these villages, the fishers cited Monção and Melgaço as the regions where the last specimens of the sea lamprey (juveniles and adults) are found each year season. The range of migration of the sea lamprey depends on factors such as river size, the extent of appropriate spawning areas, and the scale of the river downstream of insurmountable barriers [12]. In recent decades, dams have been built near the Melgaço Council, such as the Frieira hydroelectric dam in Spain, 75 km from the Minho river mouth [56, 60]. However, the hydroelectric dams present in the Minho river were not endowed with locks to migrating species, such as the sea lamprey [60]. Therefore, the sea lamprey has only the river strip to migrate until to the Frieira dam, in Spanish territory [61]. Besides, flow variations due to the Frieira dam may also affect breeding areas of migratory species such as the sea lamprey [62]. This fact influenced the conservation of these species, which were restricted to the International portion of this Iberian river [60]. Fishers may have remembered the regions around Melgaço due to the consequences generated by the proximity of this artificial barrier.

Respondents of interviews also highlighted Melgaço (26 citations) and Monção (18 quotes) as crucial points for the spawning of sea lamprey on the Minho river. They justified that these fish species could not cross this upstream point and, therefore, were the last available spawning grounds. It is worth that there were fishers (*N* = 15) who cited areas upstream of the river as spawning points and the presence of lamprey juveniles, often in practice referring to Monção and Melgaço. In the same vein, the areas of this river that present the characteristics for the reproduction of this fish species are on the banks, between Monção and Salvaterra do Minho and downstream of the Frieira dam [63].

Fishers also referred to the Monção Region as the last river point with sea lamprey stocks (juveniles and adults; *N* = 10). In the region between Monção and Melgaço, there is also an artisanal practice called “*Pesqueiras*” performed on the sloping banks of a non-navigable stretch of the Minho river [56]. These areas may also have been cited more, as they are the places where sea lamprey is most vulnerable to poaching, which may help to diminish the relative abundance composition of this fish species. Stratoudakis et al. (2016) also emphasize the vulnerability of the upstream migration season in Portuguese rivers of anadromous species due to the presence of local poachers [8]. This fact is mainly because these areas are the likely spawning sites of the species in the Minho river. As such, poachers take advantage of these circumstances to capture the animals while they are engaged in mating activities [64].

In Portugal, commercial fishing for sea lamprey to human consumption is still a significant threat to the conservation of this anadromous fish [14]. One reason is its retail elevation value that makes this cyclostome an ideal target for professional fishers and poachers [14], who may be fishing with the use of unauthorized fishing gear or outside the authorized fishing season by official bodies. Finally, in Monção and Melgaço, there is a significant concentration of restaurants specializing in sea lamprey [65], which may also have led fishers to highlight these Minho river sites as the last point of the river with the presence of these species.

Respondents also pointed out that the adult stage sea lamprey can be found all over the river (32 citations), especially the “mouth of Caminha,” in the river Minho (*N* = 8). This fact is in line with the migration pattern found in the literature that, after the marine trophic phase, this species seeks the estuaries to adapt and continue their migration upstream to spawn [12]. This migration direction of the sea lamprey was also the most mentioned by fishers (*N* = 23). Some of the interviewed (*N* = 16) also said that lampreys could migrate back to sea. Nonetheless, fishers referred only to juvenile lampreys, which, after undergoing metamorphosis in the river, seek to feed in the open sea waters in the adult stage of life [5].

In addition to the hydroelectric dams and predatory fisheries (seven citations each) already mentioned, interviewed also considered other obstacles to the migration of sea lamprey on this frontier river. According to riverine fishers (11 citations), the forces of water currents are one of the leading causes of the delay in the upstream sea lamprey displacement. Possibly, the consequences generated by the changes in the flow regime that depend on the hydroelectric dam exploitation regime [4] may have contributed to this finding. Mateus et al. (2012) also point out that sea lampreys can postpone spawning due to insurmountable obstacles such as weirs and other possible obstructions in strong currents [15].

Understanding the limitations of lamprey movement can be extremely important in helping to reduce disturbances caused by insurmountable obstacles in the natural environment [66]. Intense amounts of energy spent by the sea lamprey to overcome passage and difficult barriers during its migration can decisively impact the reproductive success of this species [67]. There are also reports of the difficulty of locomotion of the sea lamprey due to the various obstacles in the river, especially in years of low rainfall [68]. Fishers from the Minho river in this context predominantly said that this cyclostome circumvents this situation through its swimming force (32 citations). In the same line of reasoning, to overcome arduous upstream obstacles, sea lampreys under certain circumstances may increase their gust movement rather than resort to more aggressive or more prolonged moves [66]. However, lamprey shows a relatively low aerobic swimming capacity, although its maximum swimming speed is reasonably high [66, 69].

At this stage of upstream migration, sea lamprey seeks out rocky substrates at dawn to take shelter after swimming movement during the night [58–60]. Likely, these animal species are also encouraged to return to upstream-induced migration when water is released from small hydroelectric plants on the Caima River in Portugal [68]. This ethnozoological study showed that lampreys look for places in the river to rest (seven citations), instinctively deviating from natural and artificial barriers during displacement (four citations). Fishers also share that the migratory pattern of this fish species occurs day and night—at dusk or dawn (*N* = 32), but preferably at night (*N* = 13), corroborating to the scientific literature [66, 68].

In rivers, sea lamprey larvae eventually become juvenile, and later, these post-metamorphic lampreys migrate downstream to the end of estuarine and coastal waters, where they begin to feed on blood [70]. In Portugal, the larvae reach the ideal size to switch to the juvenile stage from August to February, especially October and November [71]. Downstream migration of this animal in a particular Iberian river in Spain occurs between October and May, peaking in March [70]. Riverine fishers indicate that this migration to feed in coastal areas occurs mainly in May, November, and December, within the range suggested by Silva et al. (2013) in the Iberian Peninsula [70].

Dimorphic sexual behavior, in which different individuals have different sexes, is also present in sea lamprey species [72]. All respondents (*N* = 40) shared this same information in the sampled fishing village. Most fishers (*N* = 21) could not say precisely how fertilization occurred, as they never witnessed this behavior in the river. Nonetheless, mature male sea lampreys signal that they can fertilize when they release a pheromone with a bile acid that can attract females even over long distances [73]. This animal behavior seems to encourage females to seek male-occupied nests [74]. Only nine interviewees (*N* = 9) shared this same information that females try to look for males to perform the act of fertilization. Sub-adult lamprey migrates from the sea to the coast, lakes, and rivers so they can mature, spawn, and eventually die [75]. This death of individuals of both sexes occurs due to the depletion of accumulated reserves, deterioration of the body during migration, disruption of regulatory mechanisms, and scarcity of vital substances, as well as an agglomeration of toxic products [76]. This previously shared information about this fish species give support to data collected from fishing communities. Some respondents claim that the sea lamprey performs this migration route to the Minho river when it can reproduce (*N* = 40), dying soon after spawning season (*N* = 22).

*P. marinus* begin migration intending to breed in Portuguese rivers in mid-December, with displacement peaks between February and April and spawning peak between May and June [57, 77]. Preliminary research in a fishing community in the Douro River basin indicates that sea lamprey migration occurs between December and May [78]. There are also records of this anadromous species spawning partly in winter and spring (between early January and May) upstream [3, 6, 79]. Interviews, along similar lines, indicated this act of reproduction occurs primarily in January to June with peaks in April and May, which corroborates most of the reduction interval data already published [3, 6, 57, 77–79]. In this section, the riverine interviewees also highlight the months of April, May, and June as the period of the year that has the highest number of natural deaths of this animal in the River Minho. As expected, this information from interviews conducted in fishing villages overlaps the sea lamprey spawning period in Portuguese rivers [57, 77]. Fishers (*N* = 32) also pointed out that lampreys after the parasitic feeding phase in the marine environment return to the same river sites for spawning. They described this information not as being a specific site of the river, but because of the tendency of this anadromous species to seek places upstream of the Minho river to finish reproduction. However, there is still debate about as to whether or not there is true adult loyalty in returning to the same places where they lived as larvae, where their parents performed the spawning that generated them [61].

Studies on the human ecology about lamprey (freshwater cyclostome fish or anadromous) are still scarce. In an anthropological and ecological approach to changes affecting local people in the eastern Baltic, Danto (2018) only pointed to lamprey in his study as the target of local fisheries [24]. In northwest California, USA, there is evidence of using the seasonal calendar as an indicator of fishing time, but another parasite, the lamprey eels—*Lampetra similis* (Vladykov and Kott, 1979) [26]. In this case, the traditional knowledge of tribal fishers also allowed for a more precise prediction of fish migration for more efficient local fishing. To provide strategies for promoting the well-being and ecological sustainability of a traditional North American tribe, Long and Lake (2018) only cite the use of the pacific lamprey—*Lampetra tridentatus* (Richardson, 1836) preparation as a local ecocultural resource [25]. Through the search for understanding and engagement of past peoples, it was possible to identify the *korokoro* species—the pouched lamprey (*Geotria australis* Gray, 1851*)*, in the *whakataukī* tribal database [27]. There is also a brief account of the Māori tribes of New Zealand about the time of lamprey (Kanakana) abundance in rivers and streams predominantly in spring and autumn [80].

Lastly, this present study provided crucial data from small-scale fishers' knowledge of local artisanal fisheries, folk taxonomy, preferred habitat, migration, and reproduction of the sea lamprey in the Minho river, mostly consistent with previously published data on this species (Table 5). On the island of Marajó, on the Brazilian Amazon coast, fishers also exhibited a high knowledge about local fish useful for participatory management and preservation of this animal in this region [81]. In the same vein as our findings, Australian researchers have identified the informal knowledge of recreational fishers the Murray River as a reliable source of data for proactive local fisheries management [82]. Amazonian fishers also show reliable data on fish migration and spawning behavior in the Tapajós River [83], as well as fishers in the Minho river have shown considerable ethnozoological knowledge of sea lamprey migration and reproduction. Finally, fisher’s LEK has demonstrated that the presence of dams along rivers can generate dramatic environmental changes in this aquatic system at large temporal and spatial scales [84]. In the Minho river in Portugal, fishers also emphasized the negative consequences of artificial barriers such as dams, but in this case, for the anadromous species cycle and their adverse effects on conservation.

**Table 5:** Ethnozoology data that corroborate and refute the scientific literature

Section	Scientific literature	Ethnozoology corroborated	Ethnozoology refuted or new data
*Folk taxonomy*	Lampreia (lamprey), Lampreia do mar, Lampreia marinha, and Lampreira marinha (sea lamprey) [54]. Lampreia de touca [61].	“Lampreia (lamprey), Lampreia do mar, Lampreia marinha (sea lamprey).”	“Lampreia louca, Mouca and Sapeira.”
*Preferred habitat*	Rocky bottom [56]. Shallow water between the gravel and the sandy bottom, in calmer waters [4, 55]. From 0 to 2 meters [58]. Within ranges of 2 to 5 m, 5 to 10 m or more than 10 m in this river [58]. 2 m or more in the one western European basin [59]. In the ocean can reach up to 4099 m [10]. Shady places [7, 61].	“Rock fragments, pebbles (Seixos) and sandy bottoms.” “Sites with calmer, crystalline and shallower waters, and bank muds.” “Mainly between 0 and 4 meters, shallow-water fish.” “Ranges from 0 to 8 m mostly.” “Depth sites.*”* “Lamprey swims in deep water.” “Dark places.”	Not refuted. “Sargasso Sea and herbal branches (laurel).” Not refuted. Not refuted. Not refuted. Not refuted.
*Migration*	Swimming movement during the night [58–60]. Migration occurs mostly at night, starting at twilight and ending at dawn [61]. The sea lamprey has only the river strip to migrate until to the Frieira dam, in Spanish territory [61]. The sea lamprey can migrate to other fish as a way of saving energy resources [61]. Sea lampreys under certain circumstances may increase their gust movement rather than resort to more aggressive or more prolonged moves [59]. *- Migration to reproduce:* This species seeks the estuaries to adapt and continue their migration upstream to subsequently spawn [12]. *- Trophic migration:* The Iberian river in Spain occurs between October and May, peaking in March [70]. February and April and may extend until May and June [61].	“lamprey migrates at any time of the day, but this animal prefers the nocturnal period.” “the maximum points that the sea lamprey are the Portuguese villages of Melgaço and Monção (near to hydroelectric dams).” “The sea lamprey has a lot of strength in the tail and tail. This fish hitchhike (*Boleia*) with other fish.” “They are aggressive fins (Parasites).” “Sea lamprey bypasses the obstacles along with the migration through its swimming force.” “from the mouth of the Minho river towards the source of this river.” “the sea lamprey migrates from the sea to the Minho river when they are ready for reproduction.” “November, December, January, April, May, June.” “Mainly in November, December and May.”	“occurs according to the phases of the moon.” “according to tidal variation” “in the morning.” “The center of the moon is what makes them change.” “Ourense and Illa Fillaboa, next to river Tea, in Galicia, Spain.” “They overcome all obstacles.” “Swims in the water and crawls in the sand like a snake.” Not refuted. “July, August, and September.”
*Reproduction*	Dimorphic sexual behavior is also present in sea lamprey species [72]. Females seek the nests occupied by males when they release pheromone [73, 74]. Between Monção and Salvaterra do Minho, and downstream of the Frieira da dams [63]. Late December-early January, with a peak in February /March, extending until May/June [4]. In January to June with peaks in April and May [3, 6, 57, 77–79]. There is a hypothesis as to whether or not there is true adult loyalty in returning to the same places where they lived as larvae [61]. Sub-adult lamprey spawn, and eventually die [68].	“has different sexes (males and females).” “said females found males to reproduce.” “Lamprey spawns mainly in the fishing villages of Melgaço and Monção.” “Lamprey spawns near the hydroelectric dams, and in general areas of the upstream.” “Lampreys build their nest and breed mainly from December to June.” “adult lamprey returned to the same places where they were young or where they were roe.” “Lamprey die after reproduction.”	Not refuted. “that males found females to fertilize their eggs…” “Lamprey spawns in Valença and Vila Nova de Cerveira.” “August.” “No. The lampreys go to various sides and several rivers, and even to Porto (Douro river).” “The fact that the lamprey spawns must have to do with water.”

## Conclusion

The first ethnozoological study carried out on the Minho river in Portugal with an anadromous migrant fish from the Northern Hemisphere showed a population of older fishers with an average experience of over 30 years in these small-scale fisheries. The average income of fishers was slightly above the Portuguese minimum wage, and most of these individuals supplement their income with other local economic activities. Fishing in these villages is seasonal and practiced predominantly with a drifting trammel net called “*lampreeira*.”

Three new folk names are assigned to sea lamprey in the crucial fishing villages of the Minho river. Artisanal fishers also report that *P. marinus* has sexual dimorphism and is typically shallow water, with preferred habitat in rock fragments and sandy bottoms. The locomotion occurs more at night, and during this trajectory, this animal tries to circumvent obstacles by resting on the path and using its high swimming ability. According to the interviews, part of the life cycle occurs at sea and another part in continental waters. Fishers indicate that hydroelectric dams and predatory fishing prevent lamprey migration and are potential causes for the decline of this animal population in this frontier river. Spawning of lampreys occurs mainly upstream of the Minho river, with the subsequent death of both sexes. Lastly, the general data on migration and breeding shared by fishers were typical of a cyclostome diadromous fish of the family Petromyzontidae.

Fisher’s knowledge may be an even more practical alternative in collecting data than previously thought, especially for some species that vital interests of conservation [85], such as the migratory fish in question. This threatened and valuable fishing village knowledge of intricate biological patterns should encourage researchers and managers to begin considering, evaluating, and integrating this data source as a means of improving local fishery resource management [83]. Even in situations where fisher’s scientific parameters and estimates are different at specific points, the mere fact that they make regular informal diagnoses motivates an eminent faculty to introduce these individuals into ancillary conservation efforts to generate useful estimates [86].

In recent times, there is a tendency towards the extinction of traditional knowledge about the environment and living beings [80]. And the case of lamprey fishers in Minho river does not escape the rule, with an ever-smaller number of local people who only perform this fishing activity. It also visualizes an absence of intercultural and quantitative studies that seek to describe the phenomenon of this loss of this vital source of information [87]. Modernization, the insertion of new technologies, integration into the market economy, and the schooling increase can be constituted as causes that may misstate traditional ecological knowledge [88]. Thus, cataloging information from the fishing villages of Minho on the sea lamprey becomes essential to preserve traditional practices. Also, this activity recognizes the ingrained knowledge that fishers have about this natural resource that is highly vulnerable to external environmental influences and anthropic pressures.

The ability to provide and employ local knowledge correctly can contribute to expanding society’s resilience around an ecological system, but always remembering the continuing need for the development, testing, and updating of this data [88]. Furthermore, fisher’s knowledge that has not been validated by previously published data of the species can be carefully investigated and even generate testable hypotheses for future conservation projects [89]. Ethnozoological studies with the sea lamprey in the crucial fishing communities of the Spanish part of the Minho river are recommended and could support our data and assist in the adaptive management of this anadromous migrator in this international frontier river of enormous cultural, economic, and ecological importance in the Iberian Peninsula.

## Supplementary information

**Additional file 1.** Script of interview.

## Data Availability

Not applicable.

## Abbreviations

IC: Informed Consent LEK Local Ecological Knowledge
EFH: Essential Fish Habitats ISE International Society of Ethnobiology IUCN International Union for Conservation of Nature
