# Supplementary material for: Ethnozoological knowledge of traditional fishing villages about the anadromous sea lamprey (Petromyzon marinus) in the Minho river, Portugal
Source: J Ethnobiol Ethnomed. 2019 Dec 27;15:71. doi: 10.1186/s13002-019-0345-9 (PMC6935102; doi:10.1186/s13002-019-0345-9)
Supplement: Supplementary file 1 — Additional file 1. Script of interview. [file 13002_2019_345_MOESM1_ESM.docx]

**Additional file**

**Additional file 1:** Script of interview


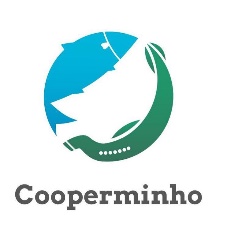


**Semi-Structured Interview Script**

******Before the interview is carried out the reading of Statement of Informed Consent (IC).

Date: ____/____/____. Number _____.

***Part I****. General data of respondent (Sociodemographic variables).*

Name (Optional): ___________________________________. Age: _________.

Fishing Village (Locality): ____________________________________.

Schooling: ( ) Illiterate. ( ) 1st Cycle (1-4 years). ( ) 2nd Cycle (5-6 years). ( ) 3rd Cycle (7-9 years), ( ) Secondary education (10-12 years). ( ) Higher education (more than 12 years).

***Part II.*** *Projective test.*

In this part, the fishers will be presented with a detailed photo of the sea lamprey - *Petromyzon marinus* Linnaeus, 1758.

( ) Fisher identified the sea lamprey. ( ) Fisher partially identified the sea lamprey. ( ) Fisher did not identify the sea lamprey.

Note: ____________________________________________________________________________________________.

***Part III.*** *Local ecological knowledge (LEK) about fishing, structure, and work equipment.*

Member of any Fishing Association? ( ) Yes . ( ) No.

Which?__________________________________________________________________. Time? __________________.

Fishing currently? ( ) Yes . ( ) No. Retired? ( ) Yes . ( ) No.

Fishing experience so far (or past): _____________________.

Income only from fishing? ( ) Yes . ( ) No. What other activity (s)?____________________________________.

Income (average)? _______________.

Lamprey Capture Season (months): __________________________________________________.

Fishing frequency during lamprey season: ( ) 1-2x per week. ( ) 3-4x per week. ( ) 5-6x per week.

( ) 7x per week.

Fishing frequency per day during the catching season: ____ hours/day.

Main fishing times: ____________________________.

What fishing gear do you use? _______________________________.

Do you use a boat? What type? _______________________________________.

Size of the boat (m): ________________________________________.

N fisher boat for lamprey fishing: ____________.

| ***Part IV:*** *Ethnozoology about the Sea Lamprey (Petromyzon marinus, Linnaeus 1758) – in the Minho River, Portugal.* | |
| --- | --- |
| ***Ethnotaxonomy (Folk name in Minho River)***  1- Adult?  2- Juveniles or larvae? |  |
| ***Habitat***  1 - Where does it live, preferably in general (Types of funds)?  2- Depth range for sea lamprey (cm or m)? (Depth).  3- In which habitat do we find larvae and juveniles of sea lamprey? (Point of the river or sea / Parish).  4- In what habitat do we find adult sea lamprey - more significant? (Point of the river or sea / Parish). |  |
| ***Reproduction***  1- Where does the sea lamprey breed? (Parish). What habitat do they spawn in Rio Minho? (Point of the river).  2- Do sea lampreys have different sexes?  3- In what months of the year does the sea lamprey build it is to nest and breed?  4- How does reproduction happen? Who is looking for who to reproduce (male or female)?  5- Do adults go back to the same places where they were young or spawned?  6- When and how do they die? Months? What happens to the male? And the female? |  |
| ***Migration / Displacement***  1- When the sea lamprey can reproduce, it goes towards to river or sea?  2- How does the lamprey move in the river? What is the direction of the migratory lamprey movement? (upstream, downstream or lateral movements)?  3- In the Minho river, the sea lamprey can migrate to which point of the river above (km or reference point)?  4- What can delay the migration of sea lamprey by the river Minho?  5- Migrations / displacements occur at what part of the day (morning / afternoon / night)?  6- When does the migration peak for reproduction occur (months or season)?  7- When does the migration to food occur? (months or season).  8- How does the sea lamprey overcome natural obstacles (current forces)? |  |
